# Comparative Analysis of Er:YAG and Er,Cr:YSGG Lasers on Root Debridement and Cell Attachment Versus Conventional Instrumentation Methods: A Scoping Review

**DOI:** 10.7759/cureus.81415

**Published:** 2025-03-29

**Authors:** E. Ecem Aydin, Ulku Baser, Funda Yalcin, Gokce Aykol Sahin

**Affiliations:** 1 Periodontology, Istanbul University School of Dentistry, Istanbul, TUR; 2 Periodontology, Istanbul Okan University, Istanbul, TUR

**Keywords:** cell adhesion, lasers, periodontal debridement, periodontal ligament, scanning electron microscopy

## Abstract

Effective subgingival debridement is essential for periodontal therapy. Subgingival debridement techniques induce morphological changes that influence healing. While hand instruments and ultrasonic scalers are the gold standard, erbium lasers offer potential advantages in biocompatibility and healing. This scoping review compares the effectiveness of erbium lasers (Er,Cr:YSGG (erbium, chromium-doped yttrium-scandium-gallium-garnet) and Er:YAG (erbium-doped yttrium-aluminum-garnet)) and conventional debridement methods in calculus removal, root surface topography, and cellular attachment following root debridement, based on in vitro studies. A systematic search was conducted in PubMed and Google Scholar for English-language articles published between 2014 and 2024. Studies evaluating erbium lasers compared to conventional debridement methods were included. A total of 1224 studies were initially identified from two databases: PubMed and Google Scholar. Following the eligibility assessment, 15 in vitro studies were analyzed. All methods effectively achieved subgingival instrumentation; additionally, erbium lasers produced smear layer-free surfaces with increased porosity, which may facilitate fibroblast attachment. However, laser-treated surfaces exhibited greater roughness and crater formation compared to conventional methods, with higher energy levels contributing to thermal damage. While some studies reported residual calculus on laser-treated surfaces, others demonstrated enhanced calculus removal and minimal root substance loss. Cell attachment studies indicated that Er:YAG lasers promoted fibroblast viability and proliferation, whereas high-energy Er,Cr:YSGG applications had variable effects on biocompatibility. Erbium lasers present a promising alternative to conventional debridement methods, improving smear layer removal and cell adhesion. However, variations in laser parameters impact treatment outcomes, emphasizing the need for standardized protocols. Further in vivo and clinical studies are required to validate their long-term effectiveness in periodontal therapy.

## Introduction and background

Periodontitis is a chronic inflammatory disease with a multifactorial etiology, primarily driven by dysbiotic plaque biofilms, and characterized by the progressive destruction of periodontal tissues, leading to clinical attachment loss (CAL), alveolar bone resorption, periodontal pocket formation, and gingival inflammation, all of which can ultimately result in tooth loss [1,2].

The primary etiological factor in periodontitis is microbial dental plaque, a structurally organized biofilm embedded in a matrix derived from both bacterial and host components [2,3]. Mineralization of supragingival plaque occurs due to salivary mineral salts, while subgingival plaque mineralizes through inflammatory exudate present within periodontal pockets [4]. The fundamental goal of non-surgical periodontal therapy is to establish a biocompatible root surface by eliminating the bacterial biofilm and its by-products, thereby promoting periodontal healing [5].

Various mechanical instrumentation techniques have been employed for periodontal debridement. Hand instruments (such as curettes) provide precision and tactile control but are time-consuming and present limitations in accessing complex root anatomy (e.g., furcations, grooves, and concavities) [6]. Ultrasonic scalers, introduced in the 1960s as an alternative to hand instruments, improve efficiency in calculus removal and are classified into magnetostrictive and piezoelectric systems [7].

With advancements in technology, lasers have emerged as adjunctive or alternative tools for periodontal therapy. The interaction of lasers with biological tissues depends on various parameters, including wavelength, pulse duration, and irradiance [8]. The wavelength, which ranges between 193 nm and 10,600 nm, is particularly crucial in determining the clinical application and biological effects of different laser systems [9].

The Nd:YAG (neodymium-doped yttrium-aluminum-garnet) laser was the first to receive FDA approval for dental applications in 1990 [10]. It has been used for sulcular debridement, bacterial reduction, and smear layer removal, but its poor absorption in water limits its applicability for hard tissue procedures [10,11]. Similarly, diode lasers have been explored for periodontal treatment but are unsuitable for subgingival calculus removal and hard tissue applications due to their thermal side effects, which may interfere with periodontal healing [9,12]. The CO₂ laser, while beneficial for soft tissue surgery, can cause morphological alterations such as melting, cracking, and carbonization when applied to hard tissues [13].

In contrast, erbium lasers, particularly the Er:YAG laser, have been developed for hard tissue applications with an optimal wavelength of 2,940 nm [14]. Its built-in cooling system prevents excessive thermal damage, making it suitable for subgingival debridement and safe for both soft and hard tissues [12,15,16]. Clinical studies have demonstrated that Er:YAG laser treatment significantly reduces bleeding on probing, periodontal pocket depth, and bacterial accumulation while also promoting fibroblast attachment and periodontal regeneration [17,18]. In addition, some studies have reported that the Er:YAG laser effectively reduces bacterial accumulation in conjunction with plaque and calculus [19-21]. Similarly, the Er,Cr:YSGG (erbium, chromium-doped yttrium-scandium-gallium-garnet) laser, with a wavelength of 2,780 nm, exhibits high absorption in water and hydroxyapatite, making it effective for hard tissue applications [22-24]. Studies suggest that the Er,Cr:YSGG laser removes subgingival calculus with comparable efficacy to conventional methods while avoiding carbonization, melting, or smear layer formation [23,25].

A previous systematic review by Alfergany et al. evaluated the effectiveness of Er:YAG and Er,Cr:YSGG lasers for calculus removal and root surface modifications, concluding that erbium lasers, when used with optimal settings, could effectively eliminate residual debris while minimizing thermal effects, making Er:YAG particularly suitable for nonsurgical periodontal therapy [26]. However, the comparative effects of erbium lasers on root surface characteristics and cellular attachment remain an area of ongoing investigation, warranting further analysis.

This scoping review aims to compare the effects of subgingival debridement using erbium lasers (Er:YAG and Er,Cr:YSGG) versus conventional instrumentation methods on root surface characteristics and cellular attachment. While erbium lasers have demonstrated promising outcomes in periodontal therapy, variations in study methodologies and energy settings have led to inconsistent findings regarding their efficacy and safety. Despite the increasing use of erbium lasers in periodontal therapy, there remains a need for further research to establish standardized protocols and determine their long-term clinical outcomes. The review focuses on evaluating the extent of surface degradation, the potential for fibroblast adhesion, and the overall impact on periodontal healing and regeneration. Additionally, it examines whether erbium lasers provide any supplementary advantages in enhancing root surface biocompatibility compared to traditional mechanical debridement techniques. By systematically analyzing in vitro studies, this review seeks to clarify the role of erbium laser technology in periodontal therapy and its implications for clinical practice.

## Review

Methods

Search Strategies

This review was limited to studies published in English between 2014 and 2024, ensuring relevance to current literature. A systematic search was conducted using PubMed and Google Scholar, employing the following keywords: "Er,Cr:YSGG laser", "Er:YAG laser", "ultrasonic periodontal instrumentation", "periodontal debridement", "periodontal ligament", and "scanning electron microscopy".

This scoping review was conducted in strict adherence to the PRISMA-ScR (Preferred Reporting Items for Systematic Reviews and Meta-Analyses Extension for Scoping Reviews) guidelines, ensuring a methodologically rigorous approach to study selection, evaluation, and reporting.

Eligibility Criteria

Studies were included based on the following criteria. Only in vitro studies evaluating various root surface debridement methods were considered, specifically those comparing erbium lasers with different techniques. Eligible studies involved single-rooted human teeth extracted due to periodontitis, as well as comparisons with healthy single-rooted teeth extracted for orthodontic reasons. Studies including multi-rooted teeth were considered only if combined with single-rooted teeth extracted due to periodontitis. All articles included were indexed in PubMed and Google Scholar, with publication dates between 2014 and 2024.

Studies meeting the following exclusion criteria were not considered: in vivo studies, clinical trials, and literature reviews. Studies that did not specify laser parameters or debridement settings were also excluded. Articles published before 2014 and studies that analyzed only multi-rooted teeth, animal teeth, or non-human cells were also excluded.

Study Selection

Study selection was conducted in two stages. In the initial screening, titles and abstracts were independently reviewed by one author (E.E.A.) to determine eligibility. Articles meeting the inclusion criteria proceeded to the full-text evaluation, where selected studies were further assessed by three independent reviewers (F.Y., Ü.B., and G.A.S.) to verify accuracy and adherence to the inclusion criteria. Any discrepancies in study selection were resolved through consensus discussion among the reviewers.

Data Extraction and Synthesis

Relevant data from each included study were systematically extracted and categorized into key parameters. These included the study title, authors and year of publication, examination methods such as scanning electron microscopy (SEM), intervention type specifying the laser system or conventional method, treatment protocol detailing the debridement technique and parameters, the objective of the study, and primary outcomes related to root surface characteristics, cellular attachment, and treatment efficacy.

A qualitative synthesis was conducted to compare the effects of erbium lasers and conventional debridement methods on root surface morphology, cellular adhesion, and overall periodontal healing potential. Due to the heterogeneity of methodologies among the included studies, a meta-analysis was not performed. Instead, the findings were synthesized descriptively to highlight trends, advantages, and limitations of laser-assisted debridement compared to traditional techniques.

Results

Study Selection

**Figure 1:**
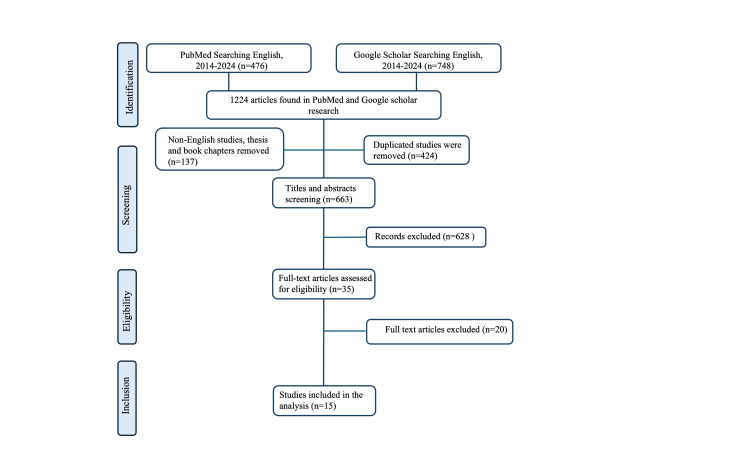
PRISMA flow diagram of studies screening and selection PRISMA: Preferred Reporting Items for Systematic Reviews and Meta-Analyses

A total of 1,224 articles were initially identified through database searches in PubMed and Google Scholar. After duplicate removal, title and abstract screening, and full-text evaluation, a total of 15 studies were included in this review. The selection process followed a systematic screening and exclusion strategy to ensure the inclusion of relevant studies (Figure 1). After applying these criteria, a total of 15 in vitro studies met the eligibility requirements and were included in the final analysis. These selected 15 studies focused on evaluating root surface alterations and cellular attachment following erbium laser treatment, providing insights into the comparative effects of Er:YAG and Er,Cr:YSGG lasers in periodontal therapy.

Study Characteristics

Surface alterations following root debridement: The characteristics of studies analyzing root surface modifications after subgingival debridement are presented in Table 1. The studies examined the effects of Er:YAG and Er,Cr:YSGG lasers compared to conventional debridement techniques (hand instruments, ultrasonic scalers) on root surface morphology. The key evaluated parameters included surface roughness (Ra values), smear layer formation and the presence of residual calculus, thermal effects and potential dentinal damage, dentinal tubule exposure, and the occurrence of structural alterations such as cracks, craters, or fractures following treatment.

**Table 1:** Effect of different periodontal instruments on root surface modification Er,Cr:YSGG: erbium, chromium-doped yttrium-scandium-gallium-garnet; Er:YAG: erbium-doped yttrium-aluminum-garnet; SEM: scanning electron microscopy

Study	Intervention	Examination Method	Parameters	Outcomes
Amid R et al., 2017 [33]	Er-YAG Laser vs. Hand Instruments	SEM Analysis Energy Dispersive X-ray Spectroscopy (EDX)	Laser 1: 100 mJ/pulse, 15 Hz, 50% air and 85% water, with 60° inclination angle, 20 s; Laser 2: 150 mJ/pulse, 15 Hz, 50% air and 85% water, with 60° inclination angle, 20 s.	The surfaces treated with lasers were rougher compared to the group where only hand instruments were used. The laser group exhibited significantly higher levels of carbon and oxygen than the hand instrument group. It was observed that the Er:YAG laser is a safe adjunct to curettes for scaling and root planing.
Belal MH and Watanabe H, 2014 [36]	Er:YAG Laser vs. CO₂ Laser	SEM Analysis	CO₂ Laser: 2H, 2W, energy density of 2.7 J/cm², 51 s Er:YAG Laser: 40 mJ/pulse energy density 14.2 J/cm² and 10 Hz, under air/water spray, 45 s.	SEM analysis confirmed more open dentinal tubules after the Er:YAG application. The CO₂ laser caused more roughness and irregularity.
Ezzat A et al., 2018 [6]	Er-Cr:YSGG Laser vs. Ultrasonic Scaler+Hand Instrument	SEM Analysis	Laser: 25 mJ, 50 Hz, 50 μs pulse duration, 20 ms pulse period, 1.25 W average power, 500 W peak power W/A:80/10 Laser+: 47 mJ, 75 Hz, 50 μs pulse duration, 3.5 W average power, 940 W peak power, W/A 40, 20. Inclination angle: 10° The time required for treatment was recorded.	Hand instruments produced smoother surfaces, whereas ultrasonic scalers showed a residual smear layer. The Er,Cr:YSGG laser exhibited superior smear layer removal but led to increased surface irregularities.
Jannath A et al., 2024 [29]	Er-Cr:YSGG Laser vs. Piezoelectric Ultrasonic Scaler	FESEM Analysis Profilometry	Ultrasonic Scaler: 30-khZ frequency Laser: 2,780 nm, 3.50 W, 75 Hz, A/W 20:40. The time was not specified.	The Er,Cr:YSGG laser demonstrated greater effectiveness in removing calculus compared to ultrasonic scalers. SEM imaging confirmed a rougher root surface with minimal thermal damage.
Karthikeyan R et al., 2020 [35]	Er:YAG Laser Nd:YAG Laser	SEM Analysis Infrared Spectroscopy	Er:YAG: 2940 nm, pulse mode 10 pulses/s, 100/200/300/400/500 mJ Nd:YAG: 1064 nm, pulse mode 1 pulse/s, 211.66, 423.33, 635, 846.66, and 1058.33 J/cm^2^. The time was not specified.	The Er:YAG laser at a lower energy level was more secure than the Nd:YAG laser in removing smear layer without revealing toxic substances. Increased energy levels correlated with greater surface alterations.
Miremadi SR et al., 2014 [30]	Er:YAG Laser vs. Ultrasonic Scaler	SEM Analysis Energy Dispersive X-ray Spectroscopy (EDX)	Laser Parameters: 60mJ, 100mJ, 160mJ and 250mJ; 2.94 µm; 10Hz; 70% water, 30% air, 20°–25° angulation angle. Treatment duration was recorded.	It was observed that the ultrasonic scaler produced a smoother surface compared to the Er:YAG laser. Carbonization, melting, or thermal changes were not detected on the laser-treated surfaces. SEM analysis confirmed that laser treatment resulted in greater dentinal tubule opening.
Naghsh N et al., 2020 [13]	Er:YAG Laser vs. CO₂ Laser vs. Ultrasonic Scaler	SEM Analysis Photomicrographs	Er:YAG: 2.94 μm, 120 mJ, air and water, 15 s, 20 Hz CO₂: 10.6 μm, 4 s, 5 cm, 3 W Hand Instrument: Until a smooth root surface was achieved. The time was not specified.	Based on the findings of the SEM analysis, the smear layer was removed from the surface of most samples in both laser groups. SEM analysis demonstrated that the Er:YAG laser was a safe and effective alternative to conventional methods by producing a biocompatible surface.
Yaghini J et al., 2015 [32]	Er:YAG vs. Ultrasonic Scaler vs. Hand Instrument	Profilometry	Er:YAG: 2940 nm, 100 mJ, 1 W, 10 Hz, water/air of 50%, 20-30° inclination angle Ultrasonic Scaler: in moderate power, under high water irrigation, inclination angle <90° The time was not specified. Hand instrument: Until all calculus was removed.	Profilometry analyses demonstrated that the laser group produced the roughest surface, whereas the ultrasonic scaler produced the smoothest.

Cellular response and attachment to treated root surfaces: Studies evaluating cell attachment and biological response following root debridement are summarized in Table 2. These studies assessed the types of cells used, including fibroblasts and periodontal ligament cells, the cell seeding density and dimensions of test specimens, and the effects of laser treatment on cellular adhesion and proliferation.

**Table 2:** Effect of different periodontal instruments on cell proliferation and attachment Er,Cr:YSGG: erbium, chromium-doped yttrium-scandium-gallium-garnet; Er:YAG: erbium-doped yttrium-aluminum-garnet; SEM: scanning electron microscopy; EDS: energy dispersive spectroscopy

Study	Intervention	Examination Method	Parameters	Outcomes
Amid R et al., 2016 [27]	Er-Cr:YSGG Laser Hand Instrument Ultrasonic Scaler	SEM Analysis MTT Assay	Laser I: 120 mJ, 10 Hz 2.79 µm Laser II: 160 mJ, 15 Hz 2.79 µm with 20-25° inclination angle; hand Instrument: 10 strokes 45/60 s for laser, 60 s for ultrasonic scaler; cell line: hGF	The laser group showed more irregularity compared to the other groups. MTT analyses indicated laser irradiation reduced the rate of proliferation and viability. SEM analysis indicated that laser irradiation adversely affected the healthy cell morphology.
Belal MH and Watanabe H, 2014 [36]	Er:YAG Laser CO₂ Laser	SEM Analysis	CO₂ Laser: 2 H, 2 W, energy density of 2.7 J/cm², 51 s; Er:YAG Laser: 40 mJ/pulse, energy density 14.2 J/cm² and 10 Hz, under air/water spray, 45 s; cell line: PDL	SEM analysis demonstrated that cell attachment was more successful in both laser groups compared to the control group. Additionally, the Er:YAG laser facilitated healthier, multilayered cell attachment with improved anastomosis.
Karam P et al., 2017 [38]	Er:YAG(ER) Nd:YAG(ND) Hand Instrument (SC) Citric Acid (CA) Antimicrobial Photodynamic Therapy (PDT)	SEM Analysis MTT Assay EDS Analysis	ER: 60 mJ, 10 pps, 10 Hz, 10 s, 2940 nm; ND: 0.5 W, 15 Hz, 10 s, 1640 nm; PDT: InGaAIP, 30 mW, 45 J/cm^2^, 30 s, 660 nm, 60 s; SC: 20 strokes; CA: citric acid (50%) + tetracycline (10%, pH 1); cell line: hGF	The Nd:YAG laser group exhibited higher cell viability and proliferation compared to the Er:YAG laser group.
Liu J et al., 2020 [34]	Er:YAG Laser	SEM Analysis Cell Culture Analysis	Group B: 25 J/cm^2^, 10 Hz, 15 s; Group C: 50 J/cm^2^, 10 Hz, 15 s; cell line: PDL	SEM analysis confirmed the absence of crack formation. The increase in energy levels enhanced surface roughness. Laser-treated surfaces had a greater number of attached fibroblasts. Additionally, variations in energy levels had no impact on fibroblast attachment.
Naghsh N et al., 2020 [13]	Er:YAG Laser vs. CO_2_ Laser vs. Ultrasonic Scaler	SEM Analysis Blood Cell Analysis	Er:YAG: 2.94 μm, 120 mJ, air and water, 15 s, 20 Hz CO₂: 10.6 μm, 4 s, 5 cm, 3 W; hand instrument: until a smooth root surface was achieved. Cell line: blood cells	Blood cell analysis results indicated that Er:YAG laser irradiation produced more biocompatible surfaces for blood cell attachment compared to CO₂ laser.
Song J et al., 2022 [31]	Er:YAG Laser vs. Nd:YAG Laser vs. Hand Instrument	SEM Analysis Cell Counting Kit-8 Assay	Er:YAG: MSP, 50 mJ, 10 Hz, 0.75 W, 2940 nm. -10° to 15° inclination angle, 10 s; Nd:YAG Laser: MSP, 15 Hz, 1.50 W, 1064 nm 30° inclination angle, 30 s; hand instrument: until a smooth root surface was achieved. 80° inclination angle. Cell line: hGF	As per the outcomes of the CCK-8 analysis, fibroblast attachment and proliferation levels were higher in the Er:YAG laser-treated group by removing calculus and reducing endotoxins. SEM analysis indicated that the Er:YAG laser produced more suitable and biocompatible surfaces compared to the Nd:YAG laser.
Talebi-Ardakani MR et al., 2017 [37]	Er:YAG Laser vs. Hand Instrument	SEM Analysis MTT Assay	Er:YAG: 2.94 μm, used at 450 mJ/pulse, 10 Hz, 94 J/cm^2^; Nd:YAG laser: 0.5 W, 15 Hz, 10 s, 1640 nm. Cell line: hGF	The MTT assay results demonstrated superior fibroblast adhesion on Er:YAG laser-treated surfaces in comparison to hand instrumentation. Increased laser energy levels correlated with enhanced roughness.

Discussion

Efficiency of Periodontal Debridement

The primary goal of subgingival debridement is to effectively remove bacterial plaque and calculus from the root surface, thereby promoting periodontal healing. Hand instruments and ultrasonic scalers are widely considered the gold standard in periodontal treatment. However, subsequent studies have demonstrated that Er,Cr:YSGG and Er:YAG lasers can serve as effective alternatives, either alone or as adjuncts to conventional instrumentation. These lasers offer advantages such as reduced treatment time, less applied force, and improved patient comfort. Nevertheless, some morphological and thermal alterations induced by laser applications pose limitations in clinical practice. This section addresses key questions regarding surface alterations and cell biology, which were introduced at the beginning of this review and further discussed throughout the analysis.

Evaluation of Er,Cr:YSGG Laser on Root Surface Modifications

Studies comparing Er,Cr:YSGG lasers with conventional techniques (ultrasonic scalers and hand instruments) have reported that the Er,Cr:YSGG laser results in greater surface roughness and irregularity [27,28]. Among the studies evaluating Er,Cr:YSGG laser efficacy, four used SEM, while one utilized profilometry as an examination method. Arora et al. highlighted that surface roughness is a critical factor influencing bacterial and cell attachment [28], whereas Amid et al. reported that excessive roughness does not contribute positively to biocompatibility [27].

In contrast, Ezzat et al. (2018) found no significant differences in root surfaces treated with an ultrasonic scaler combined with hand instrumentation versus Er,Cr:YSGG laser [6]. Additionally, Ezzat et al. noted that increasing laser energy levels correlated with greater surface damage, identifying 1.25 W as the optimal energy level for calculus removal with minimal root substance loss. Based on those study results, some calculus remnants were observed in laser-treated samples, whereas less calculus was observed in the conventional technique. However, Jannath et al. (2024) reported that the Er,Cr:YSGG laser demonstrated greater effectiveness in calculus removal compared to ultrasonic scalers, while also minimizing root surface damage [29]. In terms of root surface alterations, multiple studies indicated that Er,Cr:YSGG lasers caused melting and crater formations in contrast to ultrasonic and hand instrumentation techniques [6,28]. However, carbonization was not observed in any laser-treated samples [6]. Surfaces treated with the Er,Cr:YSGG laser exhibited minimal or no smear layer formation, which may create a more favorable environment for cell attachment and periodontal regeneration [6,28].

Evaluation of Er:YAG Laser on Root Surface Modifications

Eight studies assessed the efficacy of Er:YAG lasers on root surfaces. Among these, seven used SEM, one used profilometry, two used spectroscopy, and one used photomicrography as an examination method. Studies consistently reported that Er:YAG lasers produced significantly rougher and more irregular surface textures compared to both untreated root surfaces and those treated with conventional methods [30-33]. However, unlike other laser systems, Er:YAG did not induce melting of the root surface [30]. Increasing laser energy levels were associated with enhanced surface roughness, structural damage, crater formation, charring, and loss of root composition, while cracks or pits were not detected [30,31,34,35]. Miremadi et al. and Karthikeyan et al. identified 100 mJ as a safe and effective energy level for clinical applications. Er:YAG laser treatment also resulted in open and wider dentinal tubules with complete smear layer removal, which has been suggested to enhance biocompatibility and promote cell adhesion [13,31,34-36]. The absence of a smear layer further supports the regenerative potential of Er:YAG laser application in clinical settings. In general, erbium lasers increased surface roughness and irregularities, while ultrasonic and hand instruments resulted in smoother surfaces without thermal damage.

Evaluation of Erbium Lasers on Cell Attachment and Proliferation

Studies have shown the effects of erbium lasers on cell biology. When comparing the Er,Cr:YSGG laser to conventional methods, Amid et al. (2016) reported that the Er,Cr:YSGG laser treatment reduced cell viability and proliferation, likely due to increased surface roughness [27]. In addition, apoptotic and abnormal cells were observed in laser-treated samples.

Conversely, studies analyzing Er:YAG laser applications on different cell types have suggested positive effects on cellular behavior. Liu et al. (2020) investigated the impact of different Er:YAG laser energy levels on human periodontal fibroblast attachment and found that laser treatment enhanced cell viability and proliferation [34]. No significant differences were observed between 25 mJ and 50 mJ energy levels, with both laser groups demonstrating a higher number of viable cells compared to untreated controls. Similarly, Belal and Watanabe compared the effects of Er:YAG and CO_2_ lasers on cell viability and proliferation, reporting that Er:YAG-treated surfaces were almost entirely covered by healthy cells [36]. The presence of anastomoses and overlapping cell layers further highlighted the superior regenerative potential of Er:YAG lasers.

In a study by Naghsh et al. (2020), blood cell adhesion values were evaluated on laser-treated and conventionally (hand instrument) treated surfaces [13]. The findings suggested that Er:YAG lasers created a favorable surface for blood cell adhesion and fibrin network formation, indicating that they could be a safe and effective alternative to conventional methods.

Multiple studies have also assessed the effects of Er:YAG lasers on human gingival fibroblast cells. When comparing hand instruments to Er:YAG laser-treated surfaces, the Er:YAG laser demonstrated superior cell viability and adhesion, which was attributed to surface roughness [37]. Song et al. (2022) further analyzed human gingival fibroblast activity on root surfaces treated with various methods, reporting that toxin accumulation was more prominent in hand-instrument-treated samples [31]. The study also found that Er:YAG laser treatment enhanced cell adhesion and proliferation, particularly in specimens treated with a combination of Er:YAG and Nd:YAG lasers. SEM examination confirmed increased pseudopodia and cellular adherence in the Er:YAG laser group, reinforcing its potential as an effective hard-tissue treatment option. A study conducted by Karam et al. in 2017 concluded that the Nd:YAG laser exerted a more favorable influence on cell viability and proliferation compared to the Er:YAG laser [38].

While some studies reported negative effects of erbium lasers on healing and regeneration, the majority demonstrated a positive influence on cellular activity, particularly in enhancing cell viability and attachment.

## Conclusions

The findings of this review suggest that all examined treatment modalities are effective for subgingival debridement. Erbium lasers demonstrated minimal residual calculus, highlighting their potential in non-surgical periodontal therapy. While ultrasonic scalers were associated with more frequent surface cracks, erbium lasers primarily induced crater formation. Hand instruments created the smoothest surfaces, followed by ultrasonic scalers, whereas erbium lasers generated rougher, smear layer-free surfaces.

Laser energy levels played a critical role in surface damage, with increasing energy levels correlating with greater alterations. Conventional methods left a smear layer, while erbium lasers produced smear-free, biocompatible surfaces with potential bactericidal effects. Regarding cell adhesion and proliferation, erbium lasers generally enhanced cellular activity, although results varied. Given that these findings are based on in vitro studies, while some studies proposed certain energy parameters as potentially optimal, further research is required to establish standardized and clinically validated laser settings for periodontal therapy.
